# Clinical Characteristics of Headache in Multiple Sclerosis Patients: A Cross-Sectional Study

**DOI:** 10.3390/jcm12103518

**Published:** 2023-05-17

**Authors:** Iwona Rościszewska-Żukowska, Sabina Galiniak, Halina Bartosik-Psujek

**Affiliations:** 1 St. Jadwiga Queen Clinical Hospital No. 2, Lwowska 60, 35-301 Rzeszow, Poland; 2 Institute of Medical Sciences, Medical College, Rzeszow University, Warzywna 1a, 35-310 Rzeszow, Poland

**Keywords:** headache, migraine, tension-type headache, multiple sclerosis

## Abstract

Primary headaches are known to be associated with multiple sclerosis (MS), but previous studies concerning this relationship are not conclusive. Nowadays, there are no studies assessing the prevalence of headaches in Polish MS patients. The aim of the study was to assess the prevalence and characterise headaches in MS patients treated with disease-modifying therapies (DMTs). In a cross-sectional study of 419 consecutive RRMS patients, primary headaches were diagnosed according to the International Classification of Headache Disorders (ICHD-3) criteria. Primary headaches were observed in 236 (56%) of RRMS patients, with a higher prevalence in women (ratio of 2:1). The most common was migraine 174 (41%) (migraine with aura 80 (45%), migraine without aura 53 (30%), and probable migraine without aura 41 (23%); less frequent was tension-type headache 62 (14%). Female sex was a risk factor for migraines but not for tension-type headaches (*p* = 0.002). Migraines mostly started before MS onset (*p* = 0.023). Migraine with aura was associated with older age, longer disease duration (*p* = 0.028), and lower SDMT (*p* = 0.002). Longer DMT time was associated with migraine (*p* = 0.047), particularly migraine with aura (*p* = 0.035). Typical for migraine with aura were headaches during clinical isolated syndrome (CIS) (*p* = 0.001) and relapses (*p* = 0.025). Age and type of CIS, oligoclonal band presence, family MS history, EDSS, 9HTP, T25FW, and type of DMT did not correlate with headache. Headaches are present in more than half of MS patients treated with DMTs; migraines occur almost three times more frequently than tension-type headaches. Migraines with aura headaches during CIS and relapses are typical. Migraine in MS patients had high severity and typical migraine characteristics. DMTs had no correlation with the presence or type of headache.

## 1. Introduction

Multiple sclerosis (MS) and headache disorders, in particular migraine, have similar demographics of patients. MS is the most common chronic demyelinating autoimmune disease of the central nervous system that affects young adults, with a higher prevalence in women (2:1) [1]. Headache disorders, according to the Global Burden of Disease study, are one of the major public health clinical problems, with a high global prevalence of 52% [2,3]. Migraine is one of the most common primary headaches, with a higher prevalence in women (17% vs. 8.6%), the first cause of disability among women under 50 years of age, and an observed increase in incidence of 15.4% over the past decade [3,4,5]. Migraine in the MS population is of special interest because it has been considered an exclusion criterion for MS and is still rated as a red flag. MS and migraine with aura share a clinical presentation that could cause difficulties in differentiating MS relapse and migraine aura; therefore, migraine is the third alternative diagnosis in patients misdiagnosed with MS [6]. On the contrary, MS and migraine headaches are suggested to coexist in many young patients [7]. It remains unclear whether headache disorders are only comorbid diseases or if the diseases are causally linked. The results of previous studies on the relationship between MS and headaches are not conclusive. Several studies confirmed an association between migraine and MS, and others did not find this relationship [8,9,10]. The prevalence of headache in MS patients has been reported to be 2–49% in the meta-analysis. The pooled prevalence is estimated at 31% and depends on the continents: 24% in Asia, 43% in America, and 25% in European countries [11,12]. A recent review found that the prevalence of primary headaches among MS patients is 56%, with 27% being migraines and 10% being tension-type headaches [13]. Migraine and tension-type headaches are the most common headaches in MS patients, migraine in relapsing-remitting multiple sclerosis (RRMS), and tension-type headaches among progressive forms of MS [14]. Since the start of disease-modifying therapies (DMTs), headaches have been observed as side effects, especially with interferons and migraine headaches [15,16,17]. Other drugs did not induce headaches more frequently than the placebo group in randomised trials, although in other studies it was found that fingolimod and natalizumab can increase the frequency of migraine attacks in MS patients with migraine [17]. Although migraine is observed to be more frequent in MS patients, it is still unknown if the clinical characteristics of migraine in MS patients are different from classical symptoms. In addition, there is no data on the relationship between headache and MS-related clinical factors such as age and type of CIS, presence of oligoclonal bands, family history of MS, well-evaluated neurological conditions, and cognition impairment measured by the symbol-digital-modulation test (SDMT). To investigate these problems, we conducted a single-centre study to assess the prevalence and type of headache in patients with multiple sclerosis treated with DMT, characterise the burden of migraine, and find a relationship between migraine and MS-related clinical factors.

## 2. Materials and Methods

### 2.1. Study Design and Participants

A total of 419 consecutive patients with relapsing-remitting multiple sclerosis (RRMS) routinely admitted during DMT in one MS Centre of the St. Jadwiga Queen Clinical Hospital No. 2 in Rzeszow, Poland, between 1 June 2021, and 31 May 2022, were included in the study. All subjects were diagnosed with MS according to McDonald’s criteria and evaluated in the outpatient MS clinic [18]. Exclusion criteria were: less than 18 years, pregnant or lactating women, starting and changing DMT during the last 3 months, relapse and steroids treatment in the last 3 months to avoid impact on headache severity, history of trauma or injury to the head and/or neck, cranial or cervical vascular disorder (cerebral ischemic or haemorrhage event, vascular cranial or cervical disorder), increased or low cerebrospinal fluid pressure, hydrocephalus, brain tumour, epilepsy, and mental disorders to exclude secondary headaches, hypertension, and sinus disease in the last year to avoid interference with headache. According to the study protocol, physical and neurological examinations with the Expanded Disability Status Scale (EDSS) were performed by a trained neurologist, as well as a nine hole peg test (9HPT)—a standardised, quantitative measure of finger dexterity assessment; a timed 25 foot walk (T25FW)—a quantitative mobility and leg function test; and a written SDMT detecting cognitive impairment [19]. All participants were examined in the questionnaire. The first part for collecting demographic and clinical MS data (age, sex, educational status, professional activity, family MS history, age of clinical isolated syndrome (CIS) and diagnosis, type of CIS, presence of oligoclonal bands, disease course, and MS treatment); second headache part which checked criteria for primary headache and included clinical symptoms and history of headache (age of headache onset, disease characteristics: prodromal symptoms, pain localisation, associated symptoms, presence and aura type, frequency and intensity of migraine pain (0–10 scale) and severity of migraine evaluated by patients (severe, moderate, mild) in the last 3 months, pregnancy history, family migraine history, headache triggers, smoking, hormonal and migraine abortive, prophylaxis treatment). Primary headaches were confirmed by the neurologist—headache specialist with the 2018 ICHD-3 headache classification criteria [20]. According to these criteria, participants were diagnosed with migraine with aura, migraine without aura, probable migraine with aura, and tension-type headache.

### 2.2. Statistical Analysis

All statistical analyses were performed with the STATISTICA software package (version 13.3, StatSoft Inc., 2017, Tulsa, OK, USA). The normality of the distribution was validated using the Shapiro–Wilk test. The Mann–Whitney U test, or Kruskal–Wallis test, was used to compare differences between groups. A *p* value less than 0.05 was accepted as the significance level.

### 2.3. Ethical Approval

The study protocol was approved by the Bioethics Committee of the University of Rzeszow (protocol number 2/02/21). Written informed consent was obtained from all participants. All procedures applied during the studies involving human participants were in accordance with the ethical standards of the institutional and/or national research committees and with the Declaration of Helsinki of 1964 and its subsequent amendments or comparable ethical standards.

## 3. Results

### 3.1. Demographic and Clinical Characteristics of the Study Group

The study group (*n* = 419) consisted of 287 (68.4%) women and 132 (31.6%) men, aged 18–71 years (mean age ± SD: 40.3 ± 12.1). The clinical and demographic characteristics of the study population are presented in Table 1.

Of the patients, 236 (56.3%) had primary headaches. In the headache-positive group, female sex was significantly more predominant (172; 73%, with a ratio of 2:1) than in the non-headache group (115 (63%); *p* = 0.034). Headaches were observed to be more frequent at the secondary education level (Headache+ 85; 36.0% vs. Headache− 45; 24.6%; *p* = 0.035). Students (Headache+ 20; 8.5% vs. Headache− 18; 9.8%; *p* = 0.49), vocational (Headache+ 35; 14.8% vs. Headache− 34; 18.6%; *p* = 0.185), and high levels of education (Headache+ 91; 38.6% vs. Headache− 71; 38.8%; *p* = 0.565) were not risk factors for headaches; also, professional activity did not determine the presence of headaches. There were no differences with age, BMI, or headache prevalence (Table 1).

### 3.2. Clinical Features of MS and Headache

Headaches started more often before MS diagnosis than after MS onset (186; 78.8% vs. 50; 21.1%), with a less frequent incidence (169; 71.6% vs. 67; 28.4%). In only 28.3% of participants with the presence of headaches, the pain attacks were more frequent after MS diagnosis. The presence of headache during the first relapse (CIS) was assessed in 107 patients (45.3%) and in subsequent relapses in 99 (41.9%) participants. There were no differences in the duration of MS disease, the age of onset of clinical isolated syndrome, the type of CIS, positive oligoclonal bands, or the family history of MS due to the presence of headache. Additionally, neurological conditions measured by EDSS, 9HPT, T25FW, and SDMT were not related to the presence of headaches. The time and type of DMT do not affect headache occurrence (Table 1).

### 3.3. Type of Headache in MS Patients

Migraine was the most common type of headache in MS patients (with a ratio of 2:1 vs. the tension type of headache) and was observed in 174 (41.5%) of MS patients. Tension headache (TTH) was diagnosed in only 62 (14.8%) of MS patients. Migraine with aura (MA) was found in 80 (45.9%) participants, migraine without aura (MO) in 53 (30.4%), and probable migraine without aura (MP) in 41 (23.6%) migraine MS patients (Table 2).

### 3.4. Demographic and MS Clinical Features and Headache Type

Female sex was a relevant risk factor for migraine (136; 78.2%), not for TTH (36; 58.1%) (*p* = 0.002), irrespective of migraine type. Age was not related to migraine (39.8 ± 11.4) and TTH (39.4 ± 12.5), but it was found that older age was a predictor of only migraine with aura (42.2 ± 10.8) and not related to MO (37 ± 12.2) or MP (38.4 ± 10.5) (*p* = 0.0028).

The age of CIS and the type of CIS are not related to the prevalence of headache types. A longer duration of MS disease was a risk factor only for MO (*p* = 0.0028) (Table 2). Migraine starts before MS diagnosis, while TTH begins after MS onset (*p* = 0.023), irrespective of migraine type (Table 2). The frequency of pain before MS diagnosis was higher in migraine patients than in patients with TTH who had headaches before MS (*p* = 0.002). The presence of pain during relapses (*p* = 0.025) with CIS (*p* = 0.001) is mainly observed in migraine patients independently of migraine type and is not related to TTH. Family history of MS, oligoclonal bands, and EDSS are not related to migraine, TTH, or migraine type, while lower SDMT was observed only in migraine with aura (*p* = 0.002) (Table 2). A longer duration of DMT was not a risk factor for migraine and was not related to TTH (*p* = 0.047) or MA (*p* = 0.035). The type of DMT did not predispose to the type of headache, except ocrelizumab and TTH (*p* = 0.006) (Table 2). In migraine patients, teriflunomide treatment was more frequent in migraine with aura than in MO and MP (*p* = 0.05) (Table 2).

### 3.5. Migraine Characteristics in MS Patients

The mean monthly frequency of attack was 2.5 ± 2.1 (range: 0–13), the mean number of days of pain per month was 4 ± 3.1 (range: 0–15), and the mean migraine intensity of pain was 6.3 ± 1.9 (range: 0–10), mostly independent of migraine type. It was found that only MA (6.5 ± 2.0; range 0–10), not MO (6.4 ± 1.9; range 0–10), and MP (5.7 ± 1.7; range 0–9) were associated with a higher intensity of pain (*p* = 0.035). Most of the patients (128, 73.6%) had severe/moderate severity of migraine pain vs. mild severity of pain (46, 26.4%). The severity of severe/moderate migraine pain assessed by the patients is related to MA (66, 37.9%), not MO (33, 18.9%), and MP (39, 16.6%) (*p* = 0.009).

The clinical signs of a migraine attack with pain character and location, the impact of physical activity on pain, additional symptoms, prodromal symptoms, and triggers are shown in Table 3.

Hemilateral pain was typical for MA but not for MO and MP (*p* = 0.003). Physical activity intensifies migraine pain, particularly in MA (*p* = 0.0001). Additional symptoms were observed in 113 (64.5%) patients; the most frequent were photophobia/sonophobia and nausea, and all were strongly related to MA (photophobia/sonophobia (*p* < 0.0001), nausea (*p* = 0.009), and vomiting (*p* = 0.014)) (Table 3). The prodromal symptoms observed in most patients (112, 64.3%) were related to MA: tiredness (*p* = 0.003), attention disturbance (*p* = 0.004), stiff neck (*p* = 0.002), and photophobia/sonophobia (*p* = 0.001) (Table 3). The main migraine triggers were weather change (58.6%), stress (54.6%), and sleepless nights (41.9%); others were rare, independent of type of migraine. Oral contraceptive use, positive family history of migraine, menstrual migraine, and smoking were not related to the type of migraine (Table 3). Most patients with MS migraines receiving abortive treatment have used non-steroidal anti-inflammatory drugs (115, 66.1%); triptans were used more frequently in MA, particularly (*p* = 0.012) (Table 3). Prophylaxis treatment was rare in patients with MS with migraine (42, 24.1%), and the type of prophylaxis drugs did not differ between the types of migraine (Table 3).

## 4. Discussion

It is known that headaches are common in general and in the MS population, and migraines in general have been observed for many years as comorbidities of MS, but it is not obvious whether they are incidental or prevalent [21]. Early studies mention that the prevalence of primary headache in MS patients is low (2%); therefore, headache was suggested as an atypical syndrome and was considered a red flag in differential diagnosis [22,23,24]. In the era of DMT, in studies that mostly included immunomodulatory-treated patients, more than two-thirds of MS patients complained of headaches, mainly the most common type [25,26,27]. Headaches can play a significant role in the MS population, and their impact on the course of MS and the impact of MS on primary headaches is not yet known [28,29,30]. In clinical and radiological aspects, white matter hyperintensities are observed in even more than 1/3 of migraine women [30]. In our largest cross-sectional study and the first study among MS Polish patients, the prevalence of primary headache in MS patients is high (56%), similar to other cross-sectional studies [10,28,31,32,33] and similar to the global prevalence of headache in the general population (52%) [13]. Our results are parallel to the pool prevalence assessed in meta-analysis in cross-sectional group studies and approximately in line with case-control group studies (57%) [13]. In some previous studies, the prevalence was higher, but in most of them, the study group was much smaller (the number of participants included 34–204) [25,27,29]. We confirm previous data that headaches in MS patients are more common in women (ratio of 2:1) [34], independently of age, and more often started before MS diagnosis (78.8%). In our study group, 68% were women and 32% were men, with a ratio of 2:1. In the headache group, the ratio of women/men is greater than 2.5:1.

The prevalence of this type of headache is different between MS patients and the general population. Migraine is three times more common in MS patients than in the general population [27]. As reported by Wang et al., migraine, not TTH, is the most common primary headache in the MS population (55% vs. 20%) [13], while TTH is the most frequent in the general population (TTH 26%, migraine 12%) [5]. The high prevalence of migraine (41.5%) in our study, which is also much higher than in the general population, is consistent with previous data [5,17,26,27]. As expected, we found that migraine in MS is more than two times more common than TTH (41.5% vs. 14.8%) [25,28,30,32]. The reason for this disparity may be the differences between the study groups: MS is two to three times more common in women, and migraine is also two to three times more common in women, suggesting a sex difference that affects the neurological environment [35]. This comorbidity is particularly striking in the RRMS type. The peripheral and central nervous system inflammatory process with activated T cells and B cells, more severe in RRMS, may play a crucial role with an impact on migraine attacks in MS patients. It is supposed that cortical spreading depression during aura can lead to increased permeability of the blood-brain barrier for activated T cells, and increased cytokine levels in the brain during migraine attacks can promote inflammation in the brain [36]. Clinical studies have shown that migraine patients may also have dysregulation in their immune system with B and T cell involvement [37]. In rodents, a surprising abundance of B and T cells was reported in the meninges, with the expression of calcitonin gene-related peptide (CGRP) in B cells upon stimulation by nerve growth factor and the presence of CGRP receptors on the B and T cells. CGRP levels were much lower in inactivated cells [38]. It can be assumed that activated lymphocytes, particularly B cells, could potentially play some role in increasing CGRP, which is able to promote inflammation. This inflammation can already contribute to headaches at the time of CIS and during relapses. In previous findings, the relationship between relapse and headache was found in 19% and 27% of patients with MS, but the proportion of RRMS in these study groups was lower [28,39]. In our large study group (*n* = 419), 45% of headache patients had headaches during CIS and 41% also during subsequent relapses, with statistically significant headaches during relapse typical only for migraine in particular in MA. The presence of headaches during CIS was not associated with the type of CIS. The question remains: is headache a manifestation of symptomatic disease activity and should be classified as a secondary headache, or is it a common comorbidity with acceleration during an MS attack. It was found that in patients with other autoimmune inflammatory disorders of the central nervous system, such as neuromyelitis optica spectrum disorders and myelin oligodendrocyte glycoprotein antibody-associated disease, the frequency of headaches—among others, migraine-like headaches (not optic neuritis-related pain)—is high and can occur as a first symptom or persist during the course of the disease [40,41]. In the general population, approximately 20–30% of migraine patients also experience an aura prior to a headache [42]. In our study, MA was found in 45.9% of migraine patients. It was in line with the findings of Kister et al.; however, in our study, the prevalence of headache was much higher [27]. Female sex is a main risk factor for headache and migraine in MS patients, as previously reported [14,26,27]. We found that older age is associated only with MO. Some authors reported that older age is correlated with headaches, but the mean age in the study population was lower than in ours [26]. However, it should be noted that subclinical cognitive impairment can also be observed in patients with MS without headache and without confirmed cognitive disorders [43].

It is known to be associated with migraines of longer duration [30]. We found that particular feature in MO, as well as other MS features like EDSS, T25FW, and 9HPT that had been reported. Migraine, particularly MA, may have a negative impact on cognition, as we found a statistically lower SDMT in MA [31]. One of the arguments that migraine is a comorbidity of MS is the fact that migraine has started mostly (83%) before the onset of MS, which is consistent with previous observation, and after MS diagnosis, patients report a higher frequency of pain [44]. However, it is not consistent with previous findings in that migraine patients with MS have the same frequency and severity as typical migraine patients, and migraine disability did not differ in MS or in migraine patients in the general population [9,27].

Several DMTs have a potential headache as a side effect, in particular interferons and, to a lesser extent, natalizumab [16,45]. A systematic review showed that headaches are more common in patients with MS treated with interferons compared to the placebo group [46]. It was found that even 70% of patients without headaches prior to treatment can develop a new headache after the inclusion of interferons. Interferons can also worsen the headache independently of flu-like syndromes in half of patients with pre-existing migraine [47]. In the group treated with natalizumab (AFFIRM trial), it was observed that the frequency of headaches was observed to be higher than in the placebo group. However, other studies suggested that natalizumab did not exacerbate comorbid migraine in MS patients and supported the hypothesis that interferons might represent an important trigger for migraine worsening [48]. In other randomised, placebo-controlled phase III trials, the impact of drugs on headache associated with treatment in most DMTs was not found: teriflunomide, fingolimod, dimethyl fumarate, ocrelizumab, and clardibine. Previous research evaluating headaches in MS patients treated with DMT in clinical practise found no relationship between migraine, TTH, and any kind of DMT, particularly with interferons [25,27,29]. Our study group included participants who were all treated with DMTs, with a longer period of DMTs in migraine, particularly in MA. We did not observe any relationship between headache presence, frequency of occurrence of headache, migraine, MA, MO, and TTH with the type of DMT. We found that in patients with MS treated with ocrelizumab, more TTH was observed, but the group treated with ocrelizumab was not large (*n* = 9); more research is needed in a larger group. Additionally, MA was frequent in patients treated with teriflunomide, but we did not assess the frequency of headache before and after each inclusion of DMT.

The course of migraine in MS patients may differ from that of the general population. Our findings that the severity of migraine in our MS population is high in almost 2/3 patients, with a high monthly frequency of attack, the number of monthly pain days, and the intensity of pain, are consistent with previous data in which more severe migraines were approved in MS patients than in controls [10]. These high fluctuations cannot be explained by other triggers or other premonitory symptoms in the MS population. Some authors suggest that, in general, trigger factors are associated with multiple premonitory symptoms and can induce brain dysfunction and make patients vulnerable to migraine attacks [49]. In our MS population, at least one trigger was found in 76.2% of MS patients, and the most common reported trigger was weather change (58.6%), followed by stress (54.6%) and sleepless nights (41.9%), which is consistent with migraine triggers identified in the general population [50]. The prevalence of premonitory symptoms varies from 30 to 80% in general [51]. In our MS population, 63% of patients with the most common fatigue, attention disturbance, photophobia, neck stiffness, and yawning were reported; the first four are more related to MA, which is also consistent with the migraine population [52]. Furthermore, a primary headache, particularly a migraine, is associated with increased oxidative stress during the attack [53,54]. Due to the fact that the level of oxidative stress markers increases in the course of MS, migraine patients are particularly exposed to the adverse effects of reactive oxygen species [55,56].

Headache plays an important role not only at the time of MS diagnosis as a component of MS and a red flag in differential diagnosis but also as a stabbing type of headache that can be a symptom of MS relapse. Therefore, we suggest that the presence of headaches should be investigated during MS diagnosis, not only to avoid misdiagnosis as previously thought but also for the suspected presence of headaches during MS relapse and their negative impact on MS. A recent study by Waliszewska-Prosół et al. showed that people with migraine in Poland face similar difficulties as their peers in other countries. Moreover, it should be emphasized that migraine is undertreated in the Polish population, especially in the context of a high burden of diseases [57].

The main limitation of our study is that there is no control group and no retrospective analysis that can limit the evaluation of the outcomes of exposure to DMTs started a few years ago. Future studies should focus on the pathophysiological pathways of headache and MS and their possible common pathophysiological mechanisms and evaluate the impact of DMTs on headache in MS patients in a prospective study. Furthermore, the observation occurred during the COVID-19 pandemic, and as is known, SARS-CoV2 infections may influence the frequency and intensity of headaches [58].

## 5. Conclusions

In summary, primary headaches were present in more than half of MS patients treated with DMT, with a much higher prevalence of migraine than the tension type of headache (3:1), regardless of aura. Furthermore, female sex, type of relapsing-remitting MS, and a longer time on DMT were the main risk factors for migraine, while older age and a longer duration of the disease were the main risk factors for migraine with aura. Migraine with aura could affect cognitive impairment in MS patients measured by a lower SDMT. In patients with migraine with aura, headache attacks were typical during relapses. Migraine in MS patients had high severity and typical migraine characteristics. Finally, the type of DMT did not have a correlation with the presence or type of headache.

## Figures and Tables

**Table 1:** Clinical characteristics of the study group and patients with and without headache.

	Headache+ (*n* = 236)		Headache− (*n* = 183)		*p*	Total (*n* = 419)	
	Mean (SD)	Range	Mean (SD)	Range		Mean (SD)	Range
Disease duration from age of diagnosis (yrs)	8.5 (5.6)	0–22	9.2 (6.3)	0–28	0.440	8.8 (5.8)	0–28
Age of CIS (yrs)	29.1 (9.8)	9–60	30.4 (11.1)	6–62	0.438	29.7 (10.4)	6–62
BMI (kg/m^2^)	24.5 (4.5)	14.3–38.2	24.8 (4.4)	16.7–40.1	0.452	24.6 (4.5)	14.3–40.1
EDSS (points)	1.63 (1.0)	0–5.5	1.59 (1.1)	0–6.5	0.339	1.6 (1.1)	0–6.5
9HPT RD (s)	24.7 (14.8)	15.5–168.5	24.95 (10.7)	15–105	0.352	24.8 (13.2)	15–168.5
9HPT RND (s)	24.94 (8.2)	15.5–65	25.17 (9.9)	16.5–83.5	0.848	25.0 (9.0)	15.5–83.5
T25FW (s)	5.45 (1.7)	4–13.5	5.42 (2.3)	3.5–18.5	0.312	5.5 (2)	3.5–18.5
SDMT score	49.1 (13.6)	15–88	46.5 (13.7)	17–78	0.230	48.0 (13.7)	15–88
Time DMT (yrs)	5.7 (3.97)	0–22	6.2 (3.69)	0–16	0.149	5.9 (3.9)	0–22
	**Headache+** **(*n* = 236)**		**Headache−** **(*n* = 183)**		***p***	**Total** **(*n* = 419)**	
	***n***	**%**	**n**	**%**		***n***	**%**
CIS type							
Visual	44	16.6	32	15.8	0.783	76	18.1
Brainstem	52	19.7	29	14.3	0.118	81	19.3
Cerebellum	36	13.6	35	17.3	0.272	71	16.9
Pyramidal	49	18.6	43	21.3	0.480	92	21.9
Sensory	76	28.8	58	28.7	0.946	134	31.9
Spinal cord	7	2.9	5	2.5	0.890	12	2.9
Oligoclonal bands							
Present	140	59.3	109	59.6	0.966	249	59.4
Absent	27	11.5	30	16.4	0.384	57	13.6
Unknown	69	29.2	44	24	0.324	113	7
Family MS history	37	15.7	25	23.7	0.176	62	14.8
DMTs							
Interferons	79	33.5	61	33.3	0.976	140	33.4
Glatiramer acetate	20	8.5	17	9.3	0.771	37	8.8
Dimethyl fumarate	79	33.5	63	34.4	0.839	142	33.9
Teriflunomide	24	10.2	18	9.8	0.911	42	10
Fingolimod	16	6.8	14	7.7	0.732	30	7.2
Natalizumab	12	5.1	5	2.7	0.227	17	4.1
Ocrelizumab	5	2.1	4	2.2	0.964	9	2.1
Cladribine	1	0.4	1	0.5	0.859	2	0.5

Abbreviations: *n*, number of patients; SD, standard deviation; Headache+, headache presence; Headache−, headache absence; BMI, body mass index; EDSS, Expanded Disability Status Scale; 9HPT RD, nine hole peg test dominant hand; 9HPT RND, nine hole peg test non-dominant hand; T25FW, a timed 25 foot walk test; SDMT, Symbol Digit Modalities Test; DMT, disease-modifying treatment; and CIS, clinically isolated syndrome.

**Table 2:** Clinical MS features and type of headache.

Type of Headache *n* = 236	TTH *n* = 62		Migraine Total *n* = 174		*p*	MP *n* = 41		MO *n* = 53		MA *n* = 80		*p*
	Mean (SD)	Range	Mean (SD)	Range		Mean (SD)	Range	Mean (SD)	Range	Mean (SD)	Range	
Disease duration from age of diagnosis (yrs)	8.4 (5.6)	1–21	8.7 (5.6)	0–22	0.709	7.73 (5.5)	0–18	7.36 (5.6)	0–22	9.8 (5.4)	0–22	0.028
Age of CIS (yrs)	29.4 (10.8)	13–60	29.1 (9.4)	9–55	0.861	27.7 (8.7)	14–46	27.9 (9.7)	9–49	30.7 (9.3)	11–55	0.122
EDSS (points)	1.9 (1.3)	0–5.5	1.5 (0.9)	0–4	0.131	1.5 (0.8)	0–3.5	1.5 (0.9)	0–4	1.5 (0.8)	0–4	0.811
SDMT (points)	51.3 (16.9)	29–88	48.4 (12.2)	15–81	0.544	45.1 (10)	17–63	54.4 (13.1)	15–81	46.1 (11.4)	17–76	0.002
Time DMTs (yrs)	4.9 (3.6)	0–13	6.1 (4.1)	0–22	0.047	5.83 (4.16)	0–14	4.98 (3.97)	0–13	6.85 (4.01)	0–22	0.035
**Severity of migraine**												
Frequency of attack	NA		2.5 (2.1)	0–13	NA	2.5 (1.9)	0–8	2.4 (2.3)	0–13	2.5 (2.1)	0–10	0.712
Number of pain days per month	NA		4.0 (3.1)	0–15	NA	3.7 (2.3)	0–10	3.5 (2.6)	0–15	4.5 (3.7)	0–15	0.445
Intensity of migraine pain (range: 0–10)	NA		6.3 (1.9)	0–10	NA	5.7 (1.7)	0–9	6.4 (1.9)	0–10	6.5 (2.0)	0–10	0.035
**Type of headache** ***n* = 236**	**TTH** ***n* = 62**		**Migraine** **total** ***n* = 174**		***p***	**MP** ***n* = 41**		**MO** ***n* = 53**		**MA** ***n* = 80**		***p***
	***n***	**%**	***n***	**%**		***n***	**%**	***n***	**%**	***n***	**%**	
Start before MS diagnoses	41	66.1	145	83.3	0.023	34	82.9	44	83.0	67	83.8	0.893
Pain was more frequent before MS diagnoses	8	12.9	59	33.9	0.002	9	22.0	21	39.6	29	36.3	0.157
Pain during CIS	16	25.8	91	52.3	0.001	18	43.9	20	37.7	53	66.3	0.004
Pain during relapse	17	27.4	82	47.1	0.025	15	36.6	20	37.7	47	58.8	0.024
DMT												
Interferons	17	27.4	62	35.6	0.241	18	43.9	20	37.7	24	30	0.299
Glatiramer acetate	6	9.7	14	8.1	0.694	3	7.3	1	1.8	10	12.5	0.134
Dimethyl fumarate	19	30.5	60	34.5	0.584	12	29.2	23	43.3	25	31.2	0.258
Teriflunomide	8	12.9	16	9.2	0.409	2	4.8	2	3.7	12	15	0.05
Fingolimod	4	6.5	12	6.8	0.907	4	9.7	3	5.6	5	6.2	0.706
Natalizumab	4	6.5	8	4.6	0.571	2	4.8	3	5.6	3	3.7	0.872
Ocrelizumab	4	6.5	1	0.6	0.006	0	0	0	0	1	1.2	0.307
Cladribine	0	0	1	0.6	0.557	0	0	1	1.8	0	0	0.319

Abbreviations: *n*, number of patients; TTH, tension-type headache; MP, probable migraine without aura; MO, migraine without aura; MA, with aura; SD, standard deviation; EDSS, Expanded Disability Status Scale; SDMT, Symbol Digit Modalities Test; DMT, disease-modifying treatment; and CIS, clinically isolated syndrome.

**Table 3:** Clinical characteristics of migraine in MS patients.

Type of Migraine	Migraine Total *n* = 174		MP *n* = 41		MO *n* = 53		MA *n* = 80		*p*
	n	%	n	%	n	%	n	%	
**Time of Attack**									
Hours	99	56.9	26	14.9	33	18.9	40	22.9	0.155
Days	75	43.1	15	8.6	20	11.5	40	22.9	0.197
**Pain location**									
Bilateral	54	31	14	8	16	9.1	24	13.8	0.253
Hemilateral	110	63.2	17	9.7	37	21.2	56	32.2	0.003
Forehead/temporal	148	85.1	34	19.5	44	25.3	70	40.2	0.748
Occipital	26	14.9	7	4	9	5.1	10	5.7	0.605
**Pain character**									
Pulsating pain	102	58.6	27	15.5	30	17.2	45	25.8	0.584
Compression pain	99	56.9	14	8	33	18.9	35	20.1	0.272
**Physical activity impact on pain**	93	53.4	9	5.1	37	21.2	47	27	0.0001
**Additional symptoms**	113	64.9	17	41.5	48	90.6	73	91.3	0.0001
Nausea	72	41.3	9	5.1	27	15.5	36	45	0.009
Vomiting	24	13.7	2	1.1	13	7.4	9	11.2	0.014
Photo/sonophobia	100	57.4	12	6.9	35	20.1	63	78.7	<0.0001
**Prodromal symptoms**	112	63.4	17	41.5	31	58.5	64	80	0.0001
Fatigue	67	38.5	9	5.1	17	9.7	41	23.5	0.003
Attention disturbance	40	22.9	4	2.3	9	11.2	27	15.5	0.004
Neck stiffness	29	16.6	2	1.1	5	2.8	22	12.6	0.002
Photophobia/sonophobia	36	20.6	2	1.1	8	4.6	26	14.9	0.001
Nausea	17	9.8	1	0.6	4	2.3	12	6.9	0.071
Vision disturbance	14	8	1	0.6	3	1.7	10	5.7	0.116
Yawning	34	19.5	3	1.7	13	7.4	18	10.3	0.07
**Triggers**									
Sleepless night	73	41.9	17	9.7	21	12.0	35	20.1	0.925
Sleeping too long	23	13.2	6	3.4	10	5.7	7	4	0.210
Starving	24	13.8	4	2.3	12	6.9	8	4.6	0.067
Weather change	102	58.6	23	13.2	32	18.3	47	27	0.812
Stress	95	54.6	20	11.5	33	18.9	42	24.1	0.265
Physical activity	16	9.2	2	1.1	3	1.7	11	6.3	0.164
Wine	16	9.2	3	1.7	4	2.3	9	5.1	0.698
Chocolate	9	5.2	1	0.6	3	1.7	5	2.9	0.647
Cheese	5	2.9	0	0	2	1.1	3	1.7	0.446
Fast food	3	1.7	0	0	1	0.6	2	1.1	0.602
**Oral contraceptives**									
Never	97	55.7	23	13.2	31	17.8	43	24.7	0.964
In the past	23	13.2	6	3.4	8	4.6	9	5.1	0.579
Still	16	9.2	3	1.7	4	2.3	9	5.1	0.781
**Positive migraine family history**	59	33.9	10	5.7	21	12.0	28	16.1	0.285
**Menstrual migraine**	47	27	13	7.4	13	0.5	21	12.1	0.728
**Abortive drugs**									
Triptans	21	12.1	2	1.1	3	1.7	16	9.2	**0.012**
Non-steroidal anti-inflammatory drugs	115	66.1	25	14.3	37	21.2	53	30.4	0.499
**Prophylaxis drugs**									
Flunarizine	3	1.7	0	0	0	0	3	1.7	0.167
Metoprolol	1	0.6	0	0	0	0	1	0.6	0.555
Propranolol	15	8.6	3	1.7	1	0.6	11	6.3	0.056
Ergotaminum	1	0.6	0	0	0	0	1	0.6	0.554
Amitriptilinum	1	0.6	0	0	1	0.6	0	0	0.314
Topiramate	2	1.1	0	0	1	0.6	1	0.6	0.999
Iprazochrom	2	1.1	0	0	1	0.6	1	0.6	0.684
SSRI	19	10.9	8	4.6	2	1.1	9	5.1	0.055

## Data Availability

Data are available on request from the corresponding author.
